# Molecular characterization of EcCLP1, a new putative cathepsin L protease from *Echinococcus canadensis*[Fn FN1]

**DOI:** 10.1051/parasite/2024036

**Published:** 2024-07-09

**Authors:** Ariel Naidich, Ariana M. Gutierrez, Federico Camicia

**Affiliations:** 1 Departamento de Parasitología, Instituto Nacional de Enfermedades Infecciosas, INEI-ANLIS “Dr Carlos G. Malbrán” Av. Vélez Sársfield 563 1282 Buenos Aires Argentina; 2 Laboratorio de Toxinopatología, Centro de Patología Experimental y Aplicada, Facultad de Medicina, Universidad de Buenos Aires (UBA) José E. Uriburu 950, 5to piso 1114 Buenos Aires Argentina

**Keywords:** *Echinococcus canadensis*, *Echinococcus granulosus sensu lato*, Propeptide, Cathepsin L proteases

## Abstract

*Echinococcus granulosus sensu lato* is a platyhelminth parasite and the etiological cause of cystic echinococcosis (CE), a zoonotic and neglected disease that infects animals and humans worldwide. As a part of the biological arsenal of the parasite, cathepsin L proteases are a group of proteins that are believed to be essential for parasite penetration, immune evasion, and establishment in the tissues of the host. In this work, we have cloned and sequenced a new putative cathepsin L protease from *Echinococcus canadensis* (EcCLP1). The bioinformatic analysis suggests that EcCLP1 could be synthesized as a zymogen and activated after proteolytic cleavage. The multiple sequence alignment with other cathepsin proteases reveals important functional conserved features like a conserved active site, an N-linked glycosylation residue, a catalytic triad, an oxyanion hole, and three putative disulfide bonds. The phylogenetic analysis suggests that EcCLP1 could indeed be a cathepsin L cysteine protease from clade 1 as it grouped with cathepsins from other species in this clade. Modeling studies suggest that EcCLP1 has two domains forming a cleft where the active site is located and an occluding role for the propeptide. The transcriptomic analysis reveals different levels of cathepsin transcript expression along the different stages of the parasite life cycle. The whole-mount immunohistochemistry shows an interesting superficial punctate pattern of staining which suggests a secretory pattern of expression. The putative cathepsin L protease characterized here may represent an interesting tool for diagnostic purposes, vaccine design, or a new pharmacological target for antiparasitic intervention.

## Abstract (fr)

*Echinococcus granulosus sensu lato* est un Plathelminthe parasite et la cause étiologique de l’échinococcose kystique (EK), une maladie zoonotique et négligée qui infecte les animaux et les humains dans le monde entier. En tant que partie de l’arsenal biologique du parasite, les protéases de type cathepsine L sont un groupe de protéines considérées comme essentielles à la pénétration du parasite, l’évasion immunitaire et son établissement dans les tissus de l’hôte. Dans ce travail, nous avons cloné et séquencé une nouvelle protéase putative de type cathepsine L d’*Echinococcus canadensis* (EcCLP1). L’analyse bioinformatique suggère qu’EcCLP1 pourrait être synthétisée sous forme de zymogène et activée après clivage protéolytique. L’alignement de séquences multiples avec d’autres protéases de type cathepsine révèle d’importantes caractéristiques fonctionnelles conservées telles qu’un site actif conservé, un résidu de glycosylation lié à N, une triade catalytique, un trou oxyanion et trois liaisons disulfure putatives. L’analyse phylogénétique suggère qu’EcCLP1 pourrait en effet être une protéase de type cathepsine L du clade 1 car elle se regroupe avec les cathepsines d’autres espèces de ce clade. Les études de modélisation suggèrent qu’EcCLP1 possède deux domaines formant une fente où se trouve le site actif et un rôle d’occlusion pour le propeptide. L’analyse transcriptomique révèle différents niveaux d’expression du transcrit de la cathepsine au cours des différentes étapes du cycle de vie du parasite. L’immunohistochimie de montages entiers montre un intéressant motif de coloration ponctuée superficielle qui suggère un modèle d’expression sécrétoire. La protéase putative de type cathepsine L caractérisée ici peut représenter un outil intéressant à des fins de diagnostic, de conception de vaccins ou une nouvelle cible pharmacologique pour une intervention antiparasitaire.

## Introduction

The parasitic flatworm *Echinococcus granulosus sensu lato* (*s.l.*) is a tapeworm belonging to the class Cestoda and the Taeniidae family. Important parasitic diseases are caused globally by several species of *Echinococcus* genus (*E.* spp.) in wildlife, domestic animals, and humans. The *E. granulosus s.l.* complex, which includes *E. granulosus sensu stricto* (*s.s.*) and *Echinococcus canadensis*, is constituted by several species and most of them can potentially cause human cystic echinococcosis (formerly hydatidosis) [7, 18, 38]. Human cystic echinococcosis is one of the neglected diseases prioritized by the World Health Organization [40]. Infection in humans produces cysts usually in the liver and lungs, while other less frequent locations have also been documented [13, 14, 17, 22, 26].

Proteases are a broad group of proteolytic enzymes or peptidases, with essential functions in all living cells, such as general catabolic functions and protein processing. Belonging to this group of enzymes, cysteine proteases are lysosomal enzymes expressed by all organisms from viruses to vertebrates [32]. In *Caenorhabditis elegans* (a non-parasitic free-living nematode), Ce-CPL-1 was found, a cathepsin L protease with roles in development [15]. In parasitic helminths, proteases are essential components of the parasitic way of life because they are involved in essential functions like migration, digestion of host proteins, excystment/encystment, exsheathing, and probably, immune evasion [32]. In *Fasciola hepatica*, cysteine proteases were found to be highly immunogenic and are used as serodiagnostic markers and vaccine targets [10]. Phylogenetic analyses in this species have shown that the cathepsin L gene family expanded by a series of gene duplications and divergences that gave rise to five clades [29, 30], which are in some instances also subdivided into subclades. The significance of this expansion is unknown, but it was speculated that the divergence of the cathepsin L protease family could be important in the evolution and adaptation of the parasite to a wider host range [16]. The existence of several cathepsin L protease clades in *F. hepatica* has been revealed by phylogenetic and proteomic techniques [30]. As the life cycle of *F. hepatica* progresses, differential expression of cathepsin occurs, which allows them to effectively cleave a broad spectrum of host molecules [29]. Comprehensive biochemical investigations have revealed that cathepsins derived from this species exhibit unique substrate specificities that, while distinct, also overlap to some extent [29].

Most of the peptidases characterized at the molecular level have some sequence and structural features in common. Some of the features are the presence of a signal sequence in the amino-terminal end followed by a pro-region which is followed by the catalytic carboxy-terminal region. The peptidase is usually synthesized as a zymogen and is activated by removal of the pro-region. Even though there is a growing number of cathepsin L peptidases cloned and characterized in helminths [1, 19, 21, 25, 39], information regarding cysteine L proteases in cestodes is still scarce. However, some reports show the presence of cathepsin proteases in *Taenia solium* [1, 19, 21] and *T. crassiceps* [39], and the potential relevance of these proteases in immune evasion, nutrition, and tissue penetration [25]. Some authors have proposed that cathepsin L proteases could be used as immunodiagnostic antigens and candidate vaccine targets in *T. solium* [41]. In *Echinococcus multilocularis*, two cDNA clones encoding cysteine peptidases, named EmCLP1 and EmCLP2, were isolated in the metacestode and molecularly characterized [34]. Western blot analyses and immunohistochemistry have shown that EmCLP1 and EmCLP2 were present in *E. multilocularis* metacestodes. Recombinant EmCLP1 and EmCLP2 expressed in *Saccharomyces cerevisiae* exhibited substrate specificity against synthetic peptidyl substrates and degraded IgG, albumin, collagen, and fibronectin, which suggested those key roles in parasite–host interactions. In the present work, we have performed molecular techniques to isolate a cDNA, called EcCLP1, encoding for a putative cathepsin L-like peptidase from *E. canadensis* protoscoleces. Bioinformatic and modeling analyses suggest the existence of a propeptide with a potential role as a barrier for substrate binding, key residues for enzyme activity located in the active site cleft on a conserved tridimensional structure conformed by two domains, and close phylogenetic relationship with other cestode cathepsin L proteases. In the search for orthologs for this protease in the databases available, we observe several members for each clade, which suggests the existence of a cathepsin L family. Transcriptomic data suggest differential expression for each member of this family in each life cycle stage and finally, the immunofluorescence studies show an interesting pattern of staining that suggests a tegument secreted protein. Overall, this study suggests an important role of this protein in the penetration of host tissues and parasite development.

## Material and methods

### Ethics statement

The studies using laboratory animals adhered to authorized procedures set by the Secretaría de Ciencia y Técnica, Facultad de Ciencias Veterinarias, Universidad de La Plata, La Plata, Provincia de Buenos Aires, Argentina. The protocol titled “Estudio funcional para una cisteín proteasa de *Echinococcus granulosus* y su posible rol en la hidatidosis secundaria” reference number E36-1-13 was followed.

### Parasite material

*Echinococcus canadensis* protoscoleces were aseptically obtained through needle aspiration from hepatic hydatid cysts sourced from pigs. The parasite material was generously provided by abattoirs in the Buenos Aires province, Argentina. The livers used for extracting the parasites were obtained from animals not specifically designated for our study, and all materials were processed as part of the routine abattoir operations. Collection of samples from animals at the abattoir was conducted with the consent of local authorities. After three washes with phosphate-buffered saline (PBS), including 50 μg/mL of gentamicin to eliminate cyst wall debris, the viability of the used protoscoleces was determined through the eosin exclusion test [4]. Only samples exhibiting over 95% viability were utilized. A portion of the protoscoleces was allocated for whole-mount immunohistochemistry, another portion for cDNA synthesis, and the remaining protoscoleces were dedicated to species/genotype identification. This was achieved through sequencing a fragment of 444 bp corresponding to a part of the mitochondrial cytochrome c oxidase subunit 1 (CO1) (Supplementary Material 1), following established protocols [8, 18]. The determined species and genotype of all protoscoleces utilized in this study were identified as *E. canadensis* G7.

### RNA extraction and cDNA synthesis

RNA was extracted from *E. canadensis* protoscoleces by crushing them under liquid nitrogen and subsequently processing them with a Trizol reagent (Thermo Fisher Scientific, Waltham, MA, USA). The resulting RNA underwent treatment with RNase-Free DNase (Fermentas, Thermo Fisher Scientific), followed by ethanol precipitation. The purified RNA was then reverse transcribed using Superscript III reverse transcriptase (RT) from Life Technologies (Carlsbad, CA, USA), along with an Oligo dT primer from Invitrogen.

### Amplification and cloning of a cDNA coding for cathepsin L protease

To find the *E. canadensis* coding sequence for cathepsin L we used the coding sequence from the closest orthologue from *E. multilocularis* called EmCLP1, described by Sako et al. [34]. The primers used to amplify a complete cysteine peptidase (catalytic fragment plus prodomain) of *E. canadensis* were identical to the primers used by Sako et al. [34] and they have the following sequences: CLP1/F: 5′-GGGAATTCATTCGTCCACCTTTCCTTCA-3′ and CLP1/R: 5′-CCGTCGACTCAGTGGTGGTGGTGGTGGTGGATGAGAGGGTAGCTGGCCAT-3′. The full-length cloning of the *E. canadensis* L cathepsin was finally achieved using the mentioned CLP1/F and CLP1/R primers and the *E. canadensis* cDNA as a template. The product obtained by PCR amplification using Q51 High-Fidelity DNA Polymerase (New England Biolabs, Ipswich, MA, USA) had the expected size (~1000 base pairs, bp). The cycling parameters for the PCR were: initial denaturation step 95°, 4 min followed by 30 cycles of the following steps: melting, 95 °C, 45 s; annealing 57 °C, 45 s and extension, 72 °C, 2 min; the amplification finished with a final extension step of 10 min at 72 °C. The amplification product was visualized by agarose gel electrophoresis and Gel Red staining and the bands of interest were extracted from the gel using the QIAquick Gel Extraction Kit (QIAGEN, Hilden, Germany) and cloned into the TA TOPO cloning Kit for Sequencing (Thermo Fisher Scientific), according to manufacturer instructions. The recombinant plasmid was used for *Escherichia coli* (DH5α) transformation and the transformed bacteria was grown in an LB medium with ampicillin. The resistant colonies were then grown and used for plasmid purification using the GeneJet Plasmid miniprep kit (Fermentas). The recombinant plasmids were then sequenced using an Applied Biosystems Big Dye terminator kit (Applied Biosystems, Waltham, MA, USA) on an ABI 377 automated DNA sequencer. Here and thereafter, the cloned cDNA from *E. canadensis* was named EcCLP1 and deposited in the GenBank database under the accession number MH512909.1.

### Protein modeling

The tridimensional structures of translated protein sequences were made with Swiss Model (http://swissmodel.expasy.org/). The templates for modeling were the ProCathepsin L1 from *F. hepatica* (swiss model 2o6x.1) [36] as a template for the complete zymogen (with the propeptide) and for the mature protease the crystal structure of pro cathepsin L from *Homo sapiens* (swiss model 1cs8.1) [6] according to quality features [2] and identity. All-atom coordinates were saved in PDB files to be used with the PyMOL Molecular Graphics System, Version 2.0 Schrödinger, LLC., to generate visualizations and amino acid residue identification. All homology models obtained were validated by calculating several parameters such as QMEAN Z-score, QMEANDisCo Global, and GMQE, which were calculated using the Structure Assessment Tool of SWISS-MODEL (https://swissmodel.expasy.org/assess). Finally, structural comparisons were performed to identify relevant and conserved residues in the active site.

### Alignments and phylogeny

**Figure 1:**
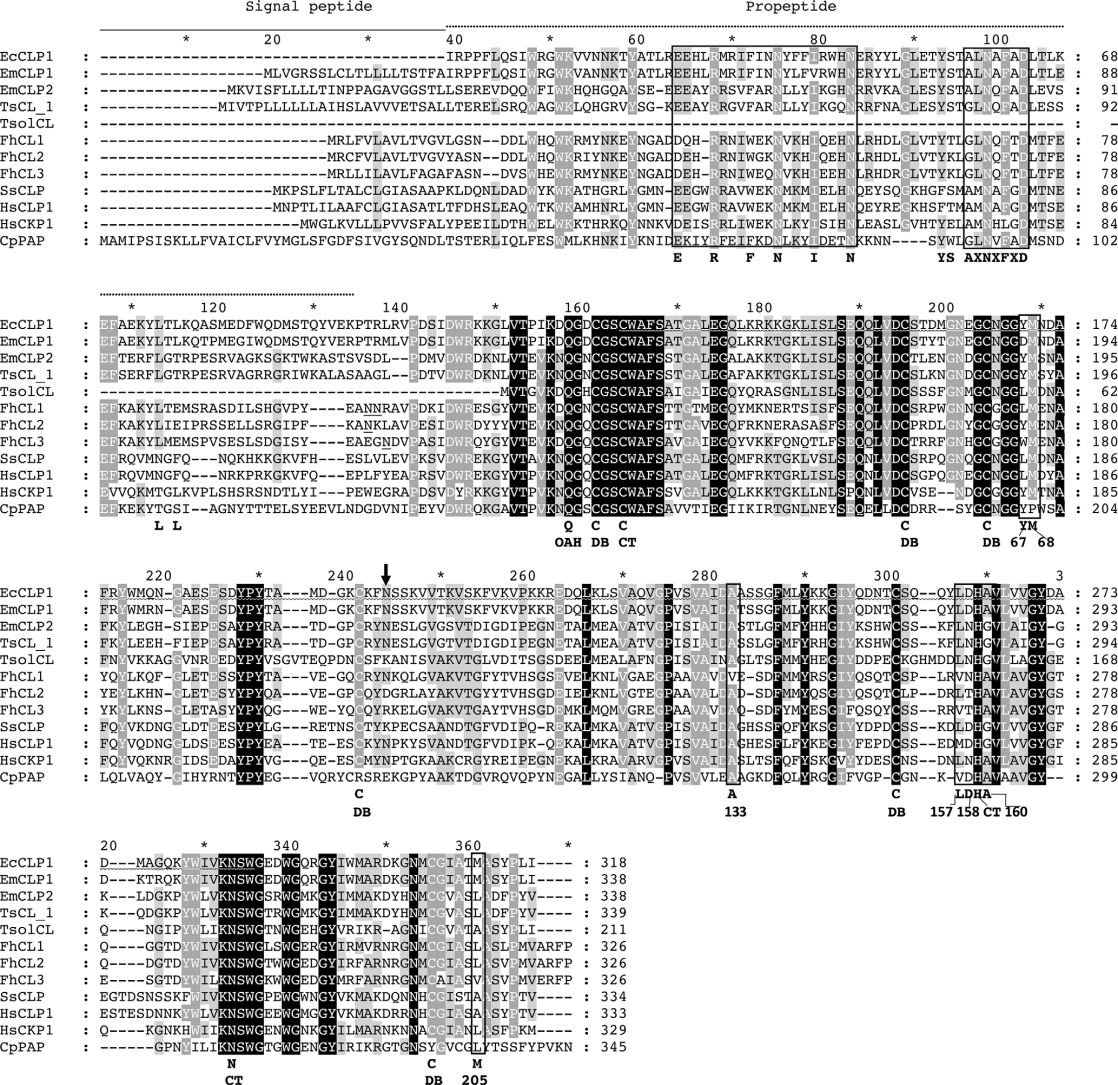
Multiple sequence alignment of the deduced amino acid sequence of EcCLP1 from *Echinococcus canadensis* with the amino acid sequence of other cathepsin proteases from several species. In the upper part of the figure, the stretch of amino acids that spans the signal peptide region is indicated by a solid line. The propeptide region is indicated by a dotted line drawn above the alignment. A N-linked glycosylation site is indicated by an arrow above the alignment. The ERFNIN and the AXNXFXD conserved motifs are indicated by squares in the propeptide region. Important residues for propeptide inhibition are indicated in the propeptide region. The asparagine residues at the juncture of the propeptide and mature enzyme, which are important for trans-activation (and propeptide removal) by asparaginyl endopeptidase, are underlined. The S2 subsite conserved residues are marked by squares and numbered according to papain numbering. CT: catalytic triad. DB: cysteine involved in a disulfide bond. OAH: oxyanion hole. EmCLP1 and EmCLP2 refer to the cathepsins L proteases from *E. multilocularis* [34] (accession numbers BAF02516.1 and BAF02517.1, respectively). The amino acid sequences of TsCL_1 and TsolCL correspond to the cathepsins L from *T. solium* [20, 21] (accession numbers AAS00027.1 and AQQ11627.1, respectively). FhCL1, FhCL2, and FhCL3 correspond to the amino acid sequences of cathepsins L1, L2, and L3 from *F. hepatica* [11, 27, 31] (accession numbers AAB41670.2, AAC47721.1 and QPX50259.1 respectively). SsCLP corresponds to the amino acid sequence of cathepsin L from *Sus scrofa* [35] (accession number Q28944 ⸳ CATL1_PIG). HsCLP1 and HsCKP1 correspond to the cathepsins L and K from *H. sapiens* (accession numbers NP_001244900.1 and NP_000387.1, respectively). CpPAP corresponds to the papain from *Carica papaya* (accession number AAB02650.1).

**Figure 2:**
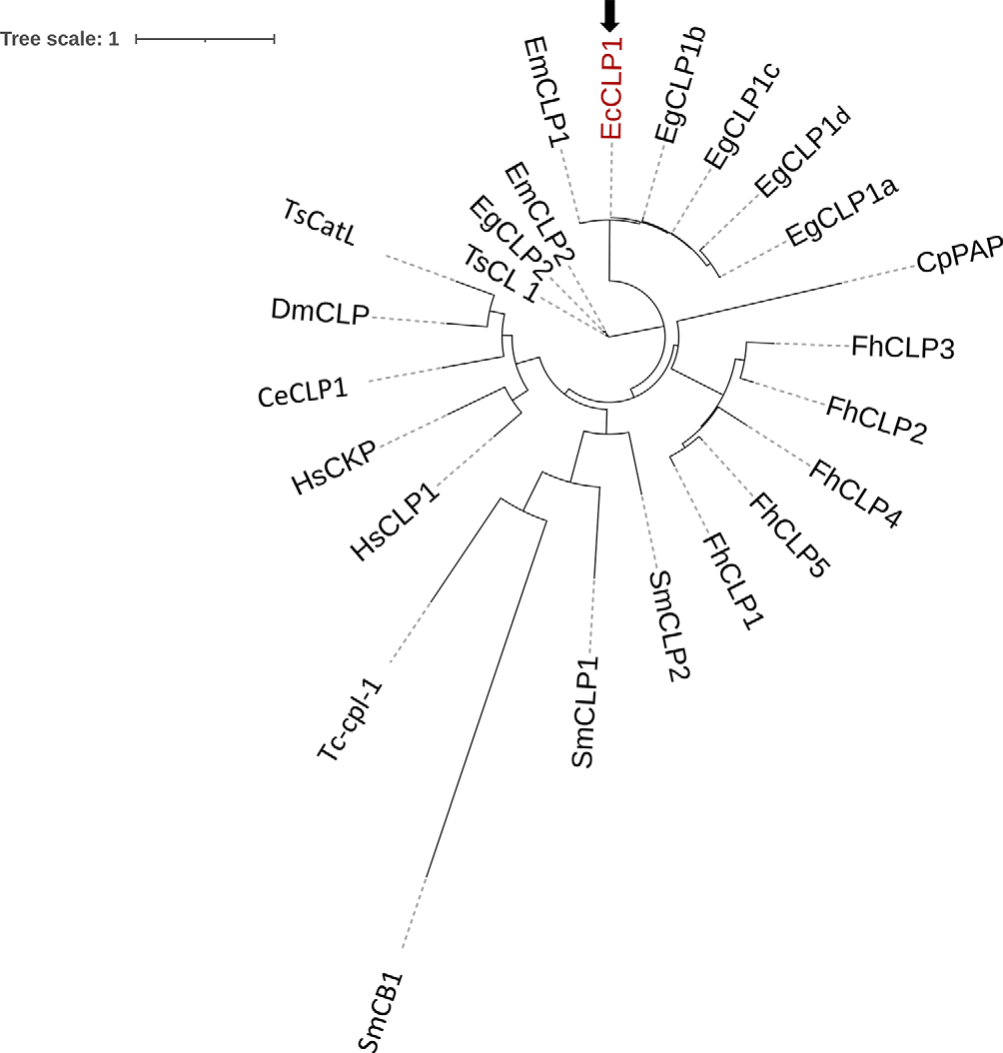
Phylogenetic tree based on the deduced amino acid sequence of EcCLP1 and cathepsin proteases from other species. The unrooted maximum likelihood tree was constructed using the phylogeny program. The black arrow indicates the EcCLP1 protease reported in this study and is marked in red letters. The accession numbers in Uniprot or GenBank databases for the species represented in the tree were the following: A0A068WJU5, A0A068WM48, A0A068WG50, A0A068WF33, U6IZB4 for the *E. granulosus s.s.* EgCLP1a, EgCLP1b, EgCLP1c, EgCLP1d and EgCLP2, respectively. BAF02516.1 and BAF02517.1 for the *E. multilocularis* EmCLP1 and EmCLP2, respectively. AAS00027.1 for the *T. solium* TsCL_1. AAB41670.2, AAC47721.1, QPX50259.1, ABZ80400.1 and AAF76330.1 for the *F. hepatica* FhCL1, FhCL2, FhCL3, FhCL4 and FhCL5, respectively. NP_001244900.1 and NP_000387.1 for the *Homo sapiens* HsCLP1 and HsCKP, respectively. AAB02650.1 for the papain CpPAP. O45734-1 for the *C. elegans* CeCLP1. AAB18345.1 for DmCLP1 or cysteine proteinase 1 from *Drosophila melanogaster*. AAC46485.1, Q26564_SCHMA_CLP2, and AAA29865.1 for SmCLP1, SmCLP2, and SmCB1, respectively from *Schistosoma mansoni*. AAC48340.1 for Tc-Cpl-1 or cathepsin L-like cysteine proteinase from *Toxocara canis*. KRY31298.1 for TsCatL or cathepsin L cysteine proteinase from *Trichinella spiralis*.

Analyses of critical residues for cathepsin L protease active site and phylogenetic characterization were performed using vertebrate and invertebrate sequences from the Peptidase C1A subfamily (MEROPS database nomenclature), composed of cysteine peptidases (CPs) similar to papain, including the mammalian CPs (cathepsins B, C, F, H, L, K, O, S, V, X and W) since high scores (very low E-values) for this family were found in the conserved domain database (https://www.ncbi.nlm.nih.gov/Structure/cdd/wrpsb.cgi). The sequences were aligned using the ClustalW program from the Expasy proteomic package (https://embnet.vital-it.ch/software/ClustalW.html). For the search of best hits, BLAST searches were performed in WormBase ParaSite (https://parasite.wormbase.org/index.html), and only sequences with at least 70% amino acid identity were chosen for the alignment. Only selected gene models were utilized for the alignment, chosen based on their optimal alignment observed through visual inspection in multiple sequence alignments. The caption of Figure 1 contains all the accession numbers for the sequences incorporated into the alignment. A phylogenetic tree was constructed using the neighbor-joining method, employing the best tree mode provided by phylogeny (https://www.phylogeny.fr/). The reliability of the tree was confirmed through bootstrap analysis, involving 500 replicates. All the sequences used for the construction of the phylogenetic tree are listed in the caption of Figure 2.

### Levels of cathepsin expression

The data about transcriptional expression levels (in RPKM, or reads per kilobase per million reads) for each cathepsin L protease clade in *E. granulosus s.s.* (G1 genotype) were obtained from Tsai [37].

### Recombinant expression

**Supplementary Figure 1:**
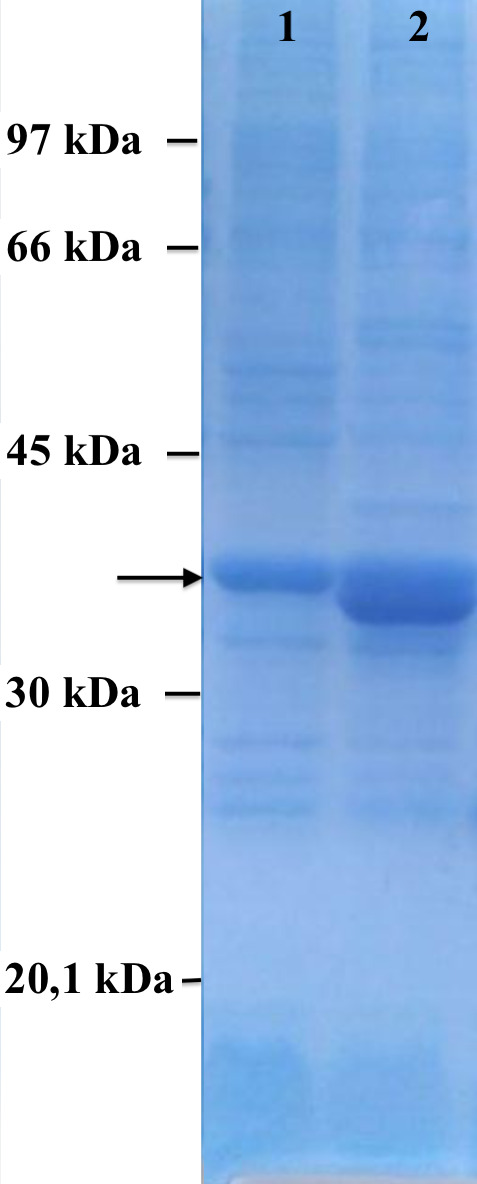
Polyacrylamide gel electrophoresis stained with Coomassie brilliant blue of bacterial cells transformed with pBAD/TOPO^®^ ThiofusionTM Expression System (ThermoFisher™) which expresses part of the EcCLP1 as a protein fused to thioredoxin (Thio-EcCLP1-his). Lane 1. Non-induced *E. coli* bacterial cells at 37 °C. Lane 2. Bacterial cells induced with 0.2% of arabinose at 37 °C. Positions of the molecular marker proteins with the corresponding masses in kilodaltons (kDa) are indicated. The arrow shows the recombinant-induced protein.

The cDNA obtained in the previous section was used as a template for PCR reaction with primers, designed for amplify the active site of eucariotic cysteine peptidases (ECP-F: 5′-CAGGGAGATTGTGGTTCGTGTTGG-3′; ECP-R: 5′-CCAACTATTCTTCACGATCCAGTA-3′) [33]. The PCR program consisted of 1 cycle at 95 °C for 4 min; 30 cycles at 95 °C during 30 s, 50 °C during 30 s, 72 °C during 30 s, and a final elongation step at 72 °C during 10 min. The PCR product containing EcCLP1 was subcloned into a pBAD/TOPO^®^ ThiofusionTM Expression System (Thermo Fisher Scientific) as a fusion protein with thioredoxin in the N-terminal end and histidine region in the C-terminal end. The recombinant construct was transformed into *Escherichia coli* BL21. The transformed bacteria were grown in LB medium with ampicillin (100 μg/mL) and colonies bearing the recombinant plasmid were selected in LB-Agar media with ampicillin. The ampicillin-resistant colonies were subsequently employed for plasmid purification, utilizing a GeneJet Plasmid miniprep kit from Fermentas. Sequencing was performed with an Applied Biosystems Big Dye terminator kit on an ABI 377 automated DNA sequencer. For induction, 200 mL of bacterial cells were treated with arabinose at a concentration of 0.2% and incubated at 37 °C for 6 h. Controls in identical conditions with non-induced bacterial cells were also run in which no arabinose was added (Supplementary Fig. 1, lane 1). Bacterial cells were pelleted at 18,621×*g* for 30 min at 4 °C and the major part of the recombinant protein expressed was detected in the induced bacterial cell pellet at 37 °C, using 15% SDS-PAGE stained with Coomassie blue (Supplementary Fig. 1, lane 2). The bacterial pellet was then resuspended in lysis buffer (Tris-HCl 20 mM, EDTA 10 mM, sucrose 10%, Triton X-100 0.1% and lysozyme 0.3 mg/mL, pH = 7.6) using 15 mL of this buffer per each gram of bacteria. The resuspended pellet was sonicated using 6 cycles of 5 s with intervals of 15 s each. The sonicated pellet was centrifuged at 16,056×*g* for 30 min at 4 °C. The pellet was resuspended in 30 mL of Tris-HCl 20 mM, sucrose 20%, Triton X-100 0.1%, and urea 2 M, pH = 7.6 and centrifuged again at 18,621×*g* for 30 min at 4 °C. Then the pellet was resuspended in a resuspension buffer containing NaH_2_PO_4_ 100 mM, Tris-HCl 10 mM, NaCl 300 mM, imidazole 20 mM, and urea 8 M, pH = 8 and centrifuged again at 21,382×*g* for 30 min at 4 °C. The sedimented bacteria were resuspended using the same buffer. The pellet was then purified using a Ni-NTA Agarose column (Invitrogen™, Cat. No. R901-15), following the instructions of the seller. Briefly, the column was washed with water to remove the methanol and equilibrated with seven volumes of resuspension buffer. The resuspended pellet was then passed in the column 3 times. The column was washed three times with resuspension buffer. The recombinant thioredoxin-EcCLP1 fusion protein with a his tag (Thio-EcCLP1-his) was eluted using the same buffer but containing imidazole at 500 mM and the eluted fractions and the flowthrough were analyzed by polyacrylamide gel electrophoresis with dodecyl sulfate (SDS-PAGE).

### Immunization and antiserum testing

**Supplementary Figure 2:**
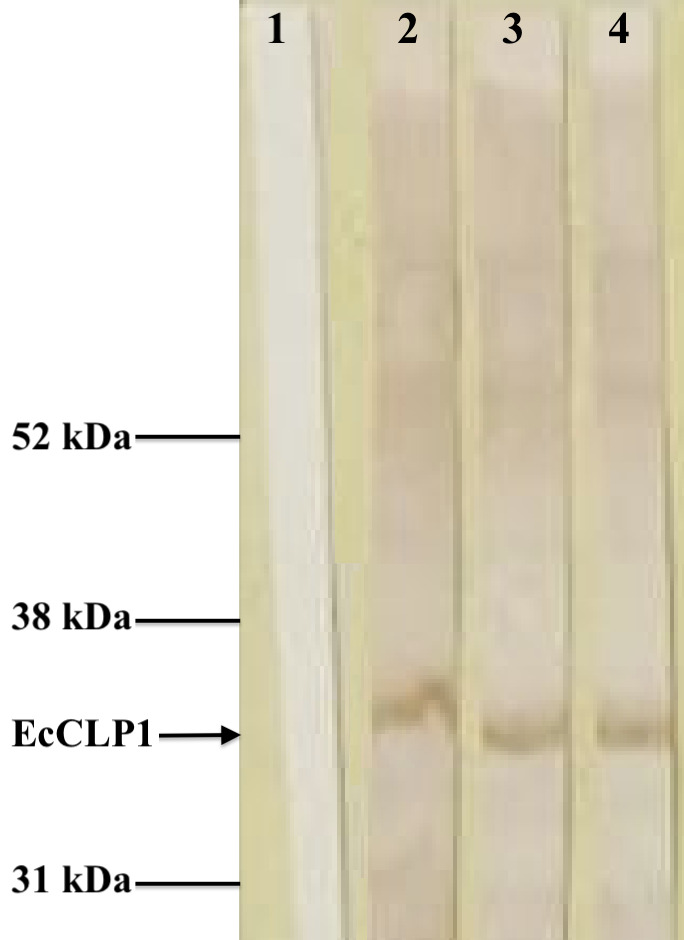
Western-blot of recombinant Thio-EcCLP1-his protein stained with rabbit hyperimmune serum against Thio-EcCLP1-his. Lane 1. Preimmune rabbit serum, diluted 1/50 in PBS-milk 1.5%. Lane 2. Hyperimmune rabbit serum diluted 1/20. Lane 3. Same hyperimmune serum as in lane 2 but diluted 1/50. Lane 4. Same hyperimmune serum as in lane 2 but diluted 1/100.

One twelve weeks old New Zealand female rabbit was immunized four times (on days 0, 30, 60, and 90) with 0.36 mg of the purified recombinant Thio-EcCLP1-his protein by intraperitoneal injection. The priming dose was administered with complete Freund’s adjuvant (Sigma-Aldrich™) and subsequent doses were formulated with incomplete Freund’s adjuvant (Sigma-Aldrich™). Thirty days after the last immunization, the rabbit was exsanguinated under general anesthesia and then euthanized by cervical dislocation. Blood was allowed to coagulate at 37 °C for 30 min and finally, serum was separated by centrifugation at 2000×*g* for 10 min. The specificity of the hyperimmune serum was determined by western blot (Supplementary Fig. 2). Briefly, 20 μg of recombinant parasite protein per well were separated on 15% polyacrylamide gels and electrotransferred to nitrocellulose membranes (BioRad™). The nitrocellulose strips were incubated with rabbit hyperimmune serum diluted at 1:100. A peroxidase-conjugated anti-rabbit IgG (Sigma™) diluted at 1:1000 was used as a secondary antibody. The bands were visualized using a diaminobenzidine reagent (ImmunoPure DAB, PIERCE, Thermo Fisher Scientific). A molecular weight marker was also run in parallel as a reference.

### Whole-mount immunohistochemistry

Freshly obtained protoscoleces of *E. canadensis* from porcine hydatid cysts were activated by treatment with pepsin in DMEM (0.05% W/V, 125 IU/mL) under acidic conditions (pH = 2) for 1 h at 37 °C with shaking (125 rpm). Then, they were allowed to sediment, washed three times with PBS, and treated with sodium taurocholate (0.2% W/V, checking that the pH remained at 7.4) for 3 h at 37 °C with shaking (125 rpm). Subsequently, the protoscoleces underwent three additional washes with PBS. Following the washes, the viability of protoscoleces was assessed using the eosin exclusion test. If they exhibited more than 95% viability, they were then fixed overnight at 4 °C with shaking in 4% paraformaldehyde. After fixation, the protoscoleces underwent three washes using PBS with 0.3% Triton X-100 (PBS-T) at room temperature with shaking. The whole-mount immunohistofluorescence protocol (WMIHF) developed by Camicia et al. [4] was then followed with slight modifications. Antibodies used were 1/50 anti-Thio-EcCLP1-his produced in rabbits, 1/50 anti-Eca-5-HT1a produced in mice [4] and preimmune rabbit serum, all of them were diluted in PBS-T. Secondary antibodies were IgG (H+L) Goat anti-rabbit conjugated to FITC from Invitrogen^TM^ and Goat anti-Mouse IgG (H+L) Secondary Antibody conjugated to Rhodamine, from Thermo Fisher Scientific^TM^, and both antibodies were used at 1/200. Specimens were finally mounted in a mix of 80% glycerol and 50 mM Tris-HCl, pH 8. The samples were observed using a Spinning Disk-TIRF-Olympus-IX83 motorized microscope equipped with a confocal module (Disk Spinning Unit). This setup was linked to a Hamamatsu Orca Flash 4 digital camera with 16-bit capability. Additionally, a confocal Zeiss LSM 880 microscope with an Airyscan detector for super-resolution was employed. The images were captured at the highest attainable resolution, specifically at 2048 × 2048 pixels. Images and stacks were viewed and processed using FIJI software (version 2.14.0/1.54f).

## Results

### EcCLP1 is a new putative cestode cathepsin L with some unusual sequence features

Based on a previous work by Sako and collaborators [34], we cloned and sequenced a putative cathepsin L protease ortholog from *E. canadensis*. The cloned sequence was identical at the translated amino acid level and in the carboxy-terminal half to the gene model EcG7_10397 from the published genome of *E. canadensis* [23] and belongs to the papain-like cysteine peptidase superfamily or IPR038765 according to the InterPro database (http://www.ebi.ac.uk/interpro/entry/InterPro/IPR038765/). The nucleotide sequence is 954 bp long with an open reading frame of 954 bp, which encodes a hypothetical protease that was called here EcCLP1. Multiple sequence alignment with other orthologous sequences encoding for cathepsins L proteases strongly suggests that the sequence found is almost complete. Except for the signal sequence which was not cloned here and is hypothetically cleaved, the sequence obtained includes the propeptide and the mature catalytic form has 318 amino acids with a predicted molecular weight of 36.48 kDa. The multiple sequence alignment of EcCLP1 with other cathepsin orthologues (Fig. 1) suggests a catalytic region of 222 amino acids in the carboxy-terminal half with a predicted molecular weight of 24.67 kDa and suggests an inhibitor propeptide of 96 amino acids in the amino-terminal end which is further cleaved in the mature form. It could be hypothesized that, based on other orthologues from *E. granulosus s.s.* and *E. multilocularis*, this sequence has a signal sequence of 20 amino acids in its amino-terminal end (Fig. 1). The sequence has one hypothetical glycosylation site in the catalytic region (Fig. 1). The multiple sequence alignment clearly shows the ERFNIN motif and the AXNXFXD motif, the latter seems to be a variant of the consensus GXNXFXD in the prosite region. Other important residues in the prosegment region are the residues YS before the AXNXFXD motif and the LXL before this motif which could be orthologous to the YK and LXE residues in the *F. hepatica* cathepsin propeptide in FhCL3 [27]. These residues could play a critical role in the binding of the propeptide to the substrate binding cleft. The asparagine residues in the juncture between the prosegment and mature enzyme domain which are conserved in cathepsins from *F. hepatica* and papain was not observed in the cestode or mammalian sequences (Fig. 1). The multiple sequence alignment shows conserved residues in the catalytic region. As a part of the S2 subsite constituting residues, it can be seen that EcCLP1 has a tyrosine in position 67 which is coincident with papain and EmCLP2, but not with EmCLP1. In position 68, EcCLP1 has a conserved methionine, which is coincident with the methionine observed in most of the cathepsin L members. Alanine is conserved in position 133 and Leucine is observed in position 157. This latter leucine is also observed in other cathepsins from cestodes and trematodes. In position 158, it is found aspartic acid, which is coincident with the residue observed in that position in EmCLP1 and HsCLP1. The alanine in position 160, is conserved in *E.* spp. and trematodes but not in other cathepsins. In position 205, the non-polar methionine is also conserved in the EmCLP1 from *E. multilocularis*, but not in the cestode EmCLP2, human HsCLP1, the three trematode FhCLPs or even in the cestode TsolCL’s cathepsins. The catalytic triad (Fig. 1) seems to be completely conserved in EcCLP1. The glutamine residue reported to form the oxyanion hole in others helminth cathepsins was also observed in the cestode cathepsin (Fig. 1). Six cysteines forming three putative disulfide bonds that are present in the catalytic domain are seen in the cestode protease (Fig. 1).

### The phylogenetic position of EcCLP1 suggests it is an L cathepsin of type 1 from cestodes

To analyze the identity of the sequence obtained, we next performed phylogenetic studies. We studied the phylogenetic position of the EcCLP1 in a phylogenetic tree composed of different members of the papain-like cysteine peptidase superfamily (http://www.ebi.ac.uk/interpro/entry/InterPro/IPR038765/). EcCLP1 is grouped in the same branch with the cathepsin of clade 1 from *E. multilocularis* or cathepsin variants from clade 1 from *E. granulosus s.s.* The topology of the tree obtained here suggests that EcCLP1 has more evolutionary proximity with cathepsins of clade 2, EmCLP2, and EgCLP2 from *E. multilocularis* and *E. granulosus s.s.*, respectively, or TsCLP1 from *T. solium* (Fig. 2) than with cathepsins from other species. The phylogenetic analysis suggests that the sequence cloned here, EcCLP1, could indeed be a cathepsin L from clade 1.

### Modeling studies suggest a conserved structure

**Figure 3:**
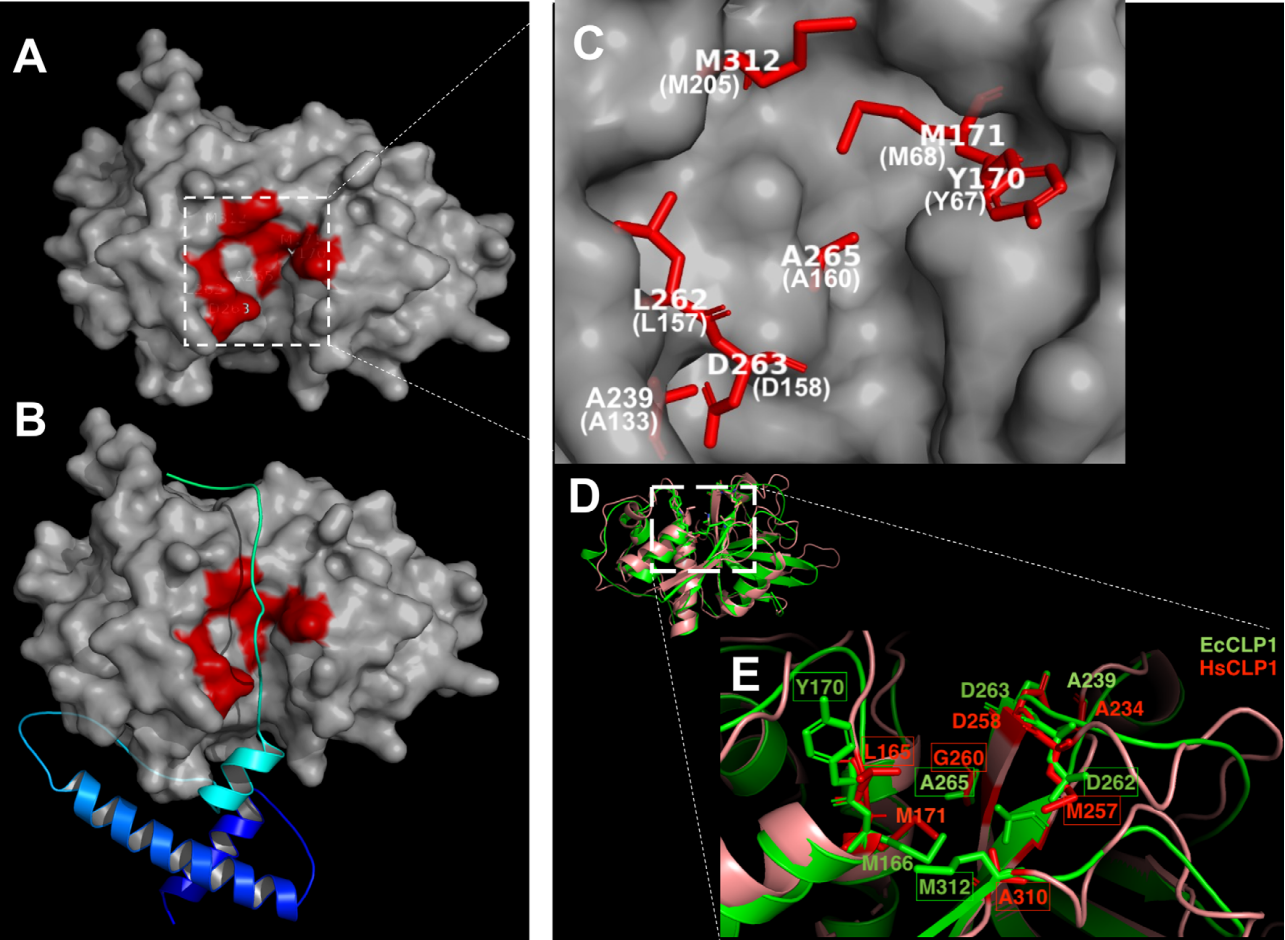
Predicted three-dimensional structures of proEcCLP1 and mature EcCLP1 and comparison with the human cathepsin. (**A**) Topological representation of the mature EcCLP1 with the residues forming the S2 subsite marked in red. (**B**) proEcCLP1 showing the propeptide in cyan and blue. (**C**) Magnified image of the model shown in (A) showing the residues forming the S2 subsite in white with papain numbering in parentheses. (**D**) Superposition of the ribbon representation of the crystal structure of human cathepsin (brown) [6] and the modeled EcCLP1 (green). (**E**) Magnified image of the active site of the cathepsins shown in (D). Important S2 residues are marked in green in parasite numbering and red in human cathepsin. Differences between both sequences are marked with boxes.

We then performed modeling studies which show a mature enzyme with two domains divided in part by a catalytic cleft (Fig. 3A). In the models obtained, the groove or catalytic cleft, which contains the active site, is transversed by the propeptide in the zymogen (Fig. 3B). The model suggests that as in other cathepsin structures, the residues tyrosine in position 170 (Y67, papain numbering in parenthesis) and leucine in position 262 (L157) are located at the entrance of the groove, while methionine in position 312 (M205) seems to be at the bottom of the groove (Fig. 3C and E). The overlapped mature EcCLP1 and human L cathepsin suggest striking similarities in the overall structures of both proteins (Fig. 3D; S1 mov); however, some important differences can be seen in the active site (Fig. 3E; Supplementary Movie).

### The cathepsins from clade 1 from *E. granulosus s.s.* have several subclades with different levels of expression during the progression of the worm life cycle

The bioinformatic analysis also suggests that cathepsins from clade 1 from *E. granulosus s.s.* probably have several variants called subclades and named a, b, c, and d.

**Figure 4:**
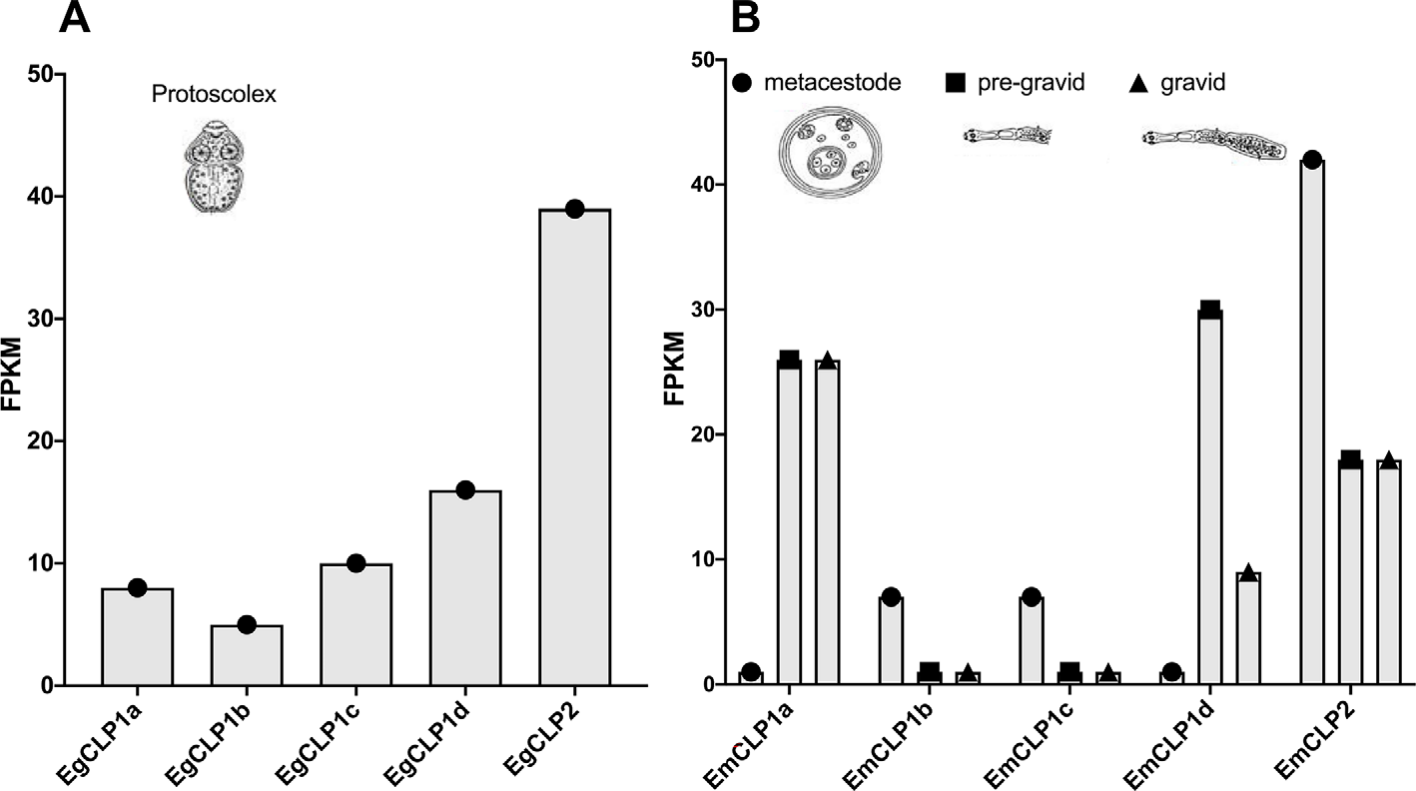
Transcriptional levels of several cathepsin L proteases in some life cycle stages from *Echinococcus.* spp. (**A**) Transcriptional levels (in FPKM levels) of the *E. granulosus s.s.* L cathepsins EgCLP1a, EgCLP1b, EgCLP1c, EgCLP1d and EgCLP2 in the protoscolex stage. (**B**) Transcriptional levels (in FPKM levels) of the *E. multilocularis* L cathepsins EmCLP1a, EmCLP1b, EmCLP1c, EmCLP1d, and EmCLP2 in the metacestode (black balls), pre-gravid (black squares) and gravid stages (black triangles). The data were obtained from Tsai et al. [37].

Figure 4, panel A (left) shows transcriptomic analysis in the protoscolex of *E. granulosus s.s.*, revealing different levels of expression of cathepsin from clade 1 (subclades a to d) and clade 2. The most relevant subclades in decreasing levels of expression are d, c, and a, followed by b with the lowest levels of transcriptional expression in the larval stage. The sum of all the transcriptional levels in FPKM for the four subclades of EgCLP1 is 39 and this level is identical to the transcriptional level of expression for clade 2. Figure 4, panel B (right) shows transcriptomic analysis of cathepsins from clade 1 and clade 2 in different stages of *E. multilocularis*. In this case, EmCLP2, EmCLP1b, and EmCLP1c have more expression than the other cathepsins in the metacestode. However, this pattern changes during development, and in the pre-adult stage, the most expressed cathepsins are EmCLP1d followed by EmCLP1a and EmCLP2. This configuration changes again during the transition to the adult in which the transcriptional levels of EmCLP1d decrease. However, the expression levels of EmCLP1a and EmCLP2 remain comparatively high.

### The whole-mount immunohistochemical detection of EcCLP1 shows a dotted and discontinuous pattern of expression in the protoscolex

**Figure 5:**
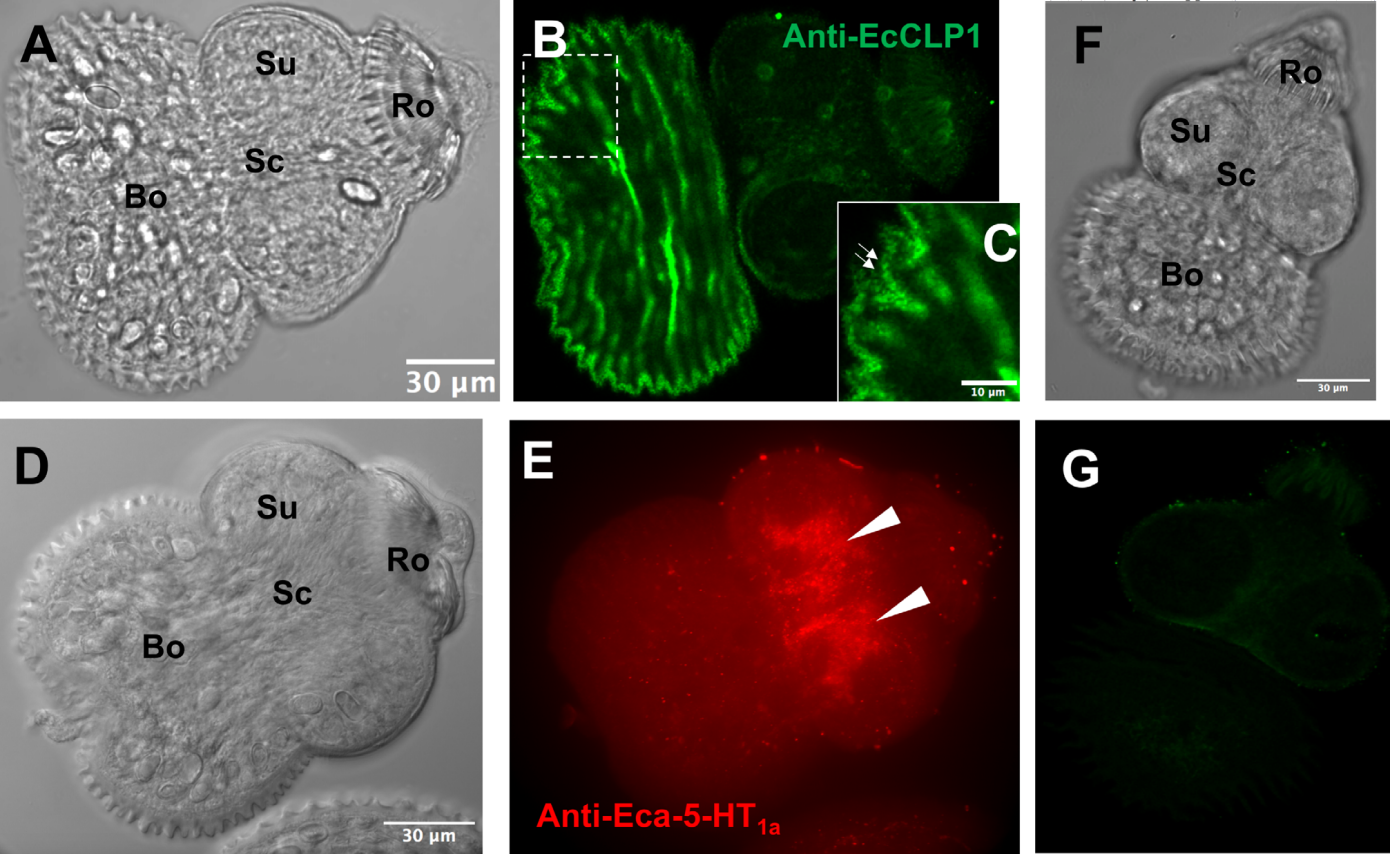
Immunolocalization of EcCLP1 in protoscoleces of *Echinococcus canadensis*. Protoscoleces were probed with anti-EcCLP1 (green) and anti-Eca-5-HT1a (red) serum and visualized by confocal microscopy. (**A**) Phase contrast view of the protoscolex shown in (B). (**B**) Fluorescent image of the protoscolex labeled with anti-EcCLP1 hyperimmune serum. Intense signals like dots were found superficially in the body region. (**C**) Magnification of the panel (B) showing a dotted pattern of staining on the surface of the tegument. (**D**) Phase contrast view of the protoscolex shown in (E). (**E**) Fluorescent image of the protoscolex labeled with anti-Eca-5-HT1a hyperimmune serum. (**F**) Phase contrast view of the protoscolex shown in (G). (**G**) Fluorescent image of the protoscolex labeled with the preimmune rabbit serum. The white arrows show the localization of Eca-5-HT1a in the cerebral ganglia in panel E or the localization of EcCLP1 in small dots in panel (C), suggesting a secretory release of the protein. Abbreviations, **Bo**: body region, **Ro**: rostellum, **Sc**: scolex region, **Su**: sucker.

We next performed a laser scanning confocal microscopy using a hyperimmune antiserum produced in rabbits against recombinant EcCLP1. Figure 5, in panels B and C (inset), shows a strong pattern of staining in the posterior part of the protoscolex. The pattern of staining of the anti-EcCLP1 is completely different from the staining observed with a hyperimmune serum against a serotonin receptor, panel E [4], suggesting that the pattern observed could be specific. The confocal scanning laser microscopy shows a rather superficial pattern of staining for EcCLP1. Another interesting feature observed in magnified pictures of the stained protoscolex is a dotted pattern of the fluorescent signal (Fig. 5, inset C). The control specimens stained with a preimmune serum showed no signal (Fig. 5 in panel G).

## Discussion

In this work, we cloned and sequenced a new putative cathepsin L protease from *E. canadensis*. This protease hypothetically has a signal sequence that was not cloned here, a propeptide, and mature catalytic regions. There are some well-conserved features in this protease, such as the ERFNIN motif and the AXNXFXD motif in the prosite region that are usually seen in other cathepsin Ls proteases, but are not seen in other proteases like cathepsin Bs [32]. In the catalytic region, EcCLP1 seems to have a conserved S2 subsite, a catalytic triad, and an oxyanion hole. However, some characteristics were observed like the presence of a tyrosine in position 67, which is more similar to EmCLP2, HsCKP1, FhCLP2, or papain than to EmCLP1. The presence of a tyrosine residue in position 67 in the mammalian cathepsin K proteases has been associated with the ability of the protease to cleave the covalently linked triple helices of native collagen [36]. The presence of leucine at position 157 and alanine at position 160, respectively makes EcCLP1 more similar to HsCKP1 and FhCLP1 than to HsCLP1. Even though leucine 157 can be seen in EmCLP1 and EmCLP2, alanine in position 160 is shared only with EmCLP1, but not with EmCLP2. The aspartate residue 158 makes EcCLP1 more similar to EmCLP1 and HsCLP1 than to other cathepsins. It is important to mention here that the presence of methionine in position 205 of EcCLP1 was not observed in cathepsins from other species in which an alanine, serine, leucine, or valine is usually seen. This residue could be characteristic of some cestode sequences from clade 1 since it was observed in the cathepsin EmCLP1 but not in EmCLP2 from *E. multilocularis*. In previous works, it was shown that the replacement of leucine by alanine at this position of FhCLP1 had a dramatic effect on enzyme specificity, being crucial to determine which P2 residues can be accommodated in the S2 pocket [36]. The presence of methionine at this position in EcCLP1 makes this protease different from the human cathepsin in this important residue and this difference could be exploited for chemotherapy. Considering the alignment all along the entire coding sequence, EcCLP1 seems to have more similarity to EmCLP1 than EmCLP2. EcCLP1 was classified as belonging to the L type and clade 1; however, the presence of the tyrosine in position 67 could indicate that EcCLP1 could have some enzymatic properties more similar to clade 2. It could be hypothesized that the presence of tyrosine in position 67, considered the “gatekeeping” position at the entrance of the S2 pocket, and the relatively larger leucine 157 at the opposite position, would make EcCLP1 more similar to FhCLP2 and HsCKP1 in terms of P2 substrate specificity than to FhCLP1. This suggests that EcCLP1 could potentially accept proline in addition to leucine (the latter is accepted by both clades, FhCLP1 and FhCLP2). However, this hypothesis, along with the influence of methionine 205 on substrate specificity, needs to be tested experimentally.

An interesting feature of EcCLP1 was the lack of asparagine residues usually seen in the juncture between the propeptide and mature enzyme domain in cathepsins sequences from trematodes. This suggests that the mechanism of trans-activation and propeptide removal described in trematodes could be different or absent in cestodes [9]. The phylogenetic analysis supported the results obtained by multiple sequence alignments and showed a clear grouping of EcCLP1 with cathepsins from clade 1. The final identity of EcCLP1 as a cathepsin protease and the assignment of EcCLP1 to its specific clade will be definitive only after functional testing of the molecule. The phylogeny shows that each cathepsin grouped better with cathepsins from the same species than with cathepsins from the same clade but of different species. In *F. hepatica*, the existence of at least 13 cathepsin genes was described [10], and cathepsins from clade 1 are subdivided into subclades. The bioinformatic analysis performed here suggests that there are at least four variants or subclades in clade 1 of *E. granulosus s.s.* and it could be hypothesized that there is a family of proteases, in a similar way as described in *F. hepatica*. The modeling studies suggest that EcCLP1 is a two-domain protease with a major groove that forms the catalytic site. The zymogen structure shows that the propeptide transverses the catalytic cleft, suggesting that it could have a role as a barrier, blocking the substrate’s access to the active site. The structure of the active site seems to be conserved with major residues like tyrosine 170 (Y67) or leucine 262 (L157) probably playing a major role at the entrance of the cleft and methionine 312 (M205) at the bottom of the cleft (papain numbering in parenthesis).

The change in the transcriptional levels of *E. granulosus s.s.* cathepsins during the parasite stage transition suggest that these proteases could play important roles during larval establishment and development. In *F. hepatica*, which was more studied than *E.* spp. in this subject, it was observed that the temporal expression and secretion of distinct members of a family of cathepsin L cysteine peptidases (FhCL) correlates with the entry and migration of the helminth pathogen in the host [28, 29]. Therefore, as infective larvae move through the intestinal wall, they release cathepsin L3 (FhCL3). In the case of juvenile parasites migrating through the liver, both FhCL1 and FhCL2 are secreted. In mature bile duct parasites, which depend on blood feeding, the predominant secretion is FhCL1, although FhCL2 is also released [28]. We wonder whether there is a similar pattern in timing and whether levels of expression of different clades and subclades of cathepsins L peptidases are differentially expressed during the life cycle progression of *Echinococcus* spp. At the time of writing, no orthologous genes to the cathepsin clades 3, 4, or 5 were found in *E. granulosus s.s.*; however, this does not necessarily mean that these clades do not exist in this genus. The transcriptomic data suggest that CLP1b and CLP1c, together with CLP2 could play important roles during early metacestode development, CLP1d in more adult advanced stages, and finally CLP1a in the adult gravid stage. Nonetheless, this assumption should be tested experimentally.

The laser scanning confocal microscopy shows an interesting superficial pattern of staining using hyperimmune serum against the Thio-EcCLP1-His recombinant fusion protein. A punctate pattern of staining on the surface of the tegument in the body of the protoscolex can be seen. In our hands, a hyperimmune serum or purified IgG antibody against thioredoxin alone did not show any staining in protoscolex sections [3], suggesting that the pattern observed could be attributed to the mature recombinant portion of EcCLP1. The punctate pattern of staining could not be seen in the histochemical staining obtained by Sako et al. [34] probably given the lower resolution of the technique employed based on cryosections stained with diaminobenzidine. The pattern obtained with a hyperimmune rabbit serum suggests that EcCLP1 is probably a secretory protein. This pattern was expected given that cathepsins were seen in the gut in other helminths [12, 15, 27]. Considering that in cestodes the digestion and absorption of nutrients is performed by the tegument, the pattern of staining obtained is compatible with such functions. Secretion of cysteine proteases was associated with virulence in other Platyhelminthes species for example in *F. hepatica*. In this latter species, cathepsin L1 (FheCL1) and cathepsin L2 (FheCL2) represent important peptidases released by infective larvae crossing the host intestinal wall, migrating stages penetrating liver tissues, and mature adult parasites residing in bile ducts. The mature parasites feed on host blood, which they ingest by puncturing the bile duct wall [36].

Immunocytochemistry using antiserum against the mature cathepsin L from *F. hepatica*, localized this protease to the gastrodermal epithelial cells showing a punctate pattern of staining and the electron microscopy revealed that the enzyme is indeed stored in secretory vesicles within these cells [5]. The fluorescent pattern of staining was similar to the punctate pattern observed here, suggesting that both cathepsins could have similar roles. In a similar way for *E. canadensis*, we suspect that EcCLP1 secretion could also play a major role in *E. canadensis* virulence; however, further experiments should be performed to test this hypothesis.

In summary, we have cloned and sequenced a new sequence that putatively encodes for a new cathepsin L cysteine protease from clade 1. The bioinformatic analysis shows the presence of several sequence residues with major roles in cathepsin function. Modeling studies suggest that EcCLP1 has a conserved structure, but important residue differences at the active site could be seen (like tyrosine 67 or methionine 205 among others) and such notable differences could be exploited pharmacologically. The phylogenetic analysis grouped the sequence obtained with other cystine L cathepsins of clade 1. The transcriptomic data suggest a particular pattern of expression of each clade (and subclade) of L cathepsins during the progression of the life cycle. Finally, the observed pattern of expression of EcCLP1 suggests that this hypothetical protein could be a secretory protein. The study of this type of protein has been useful not only for understanding the biology of the parasite, but also for diagnostic [20] and chemotherapeutic purposes [24], as well as for vaccine design [10]. Given the potential role of cathepsins in larval tissue penetration or parasite establishment, the study of cathepsins in *Echinococcus* spp. could also be of major relevance to vaccine design or finding a new target for chemotherapeutic interventions.

## Supplementary materials

The supplementary material for this article can be found at https://www.parasite-journal.org/10.1051/parasite/2024036/olm.

*Supplementary Material 1*: Sequence obtained from the mitochondrial cytochrome c oxidase subunit 1 (CO1) sequencing and primers employed for species and genotype determination of the protoscoleces utilized in this study. The genotype obtained correspond to G7 or pig strain. *Supplementary Movie 1*: Superposition of the ribbon representation of the crystal structure of human cathepsin (brown, [35]) and the modeled EcCLP1 (green). Magnified view at the active site.
