# Supplementary material for: Molecular characterization of EcCLP1, a new putative cathepsin L protease from Echinococcus canadensis
Source: Parasite. 2024 Jul 9;31:39. doi: 10.1051/parasite/2024036 (PMC11242924; doi:10.1051/parasite/2024036)
Supplement: Supplementary file 1 — Sequence obtained from the mitochondrial cytochrome c oxidase subunit 1 (CO1) sequencing and primers employed for species and genotype determination of the protoscoleces utilized in this study. The genotype obtained correspond to G7 or pig strain. [file parasite-31-39-s1.pdf]

>*Echinococcus canadensis* cytochrome c oxidase subunit 1 (COI) gene
TTTTTTGGGCATCCTGAGGTTTATGTGTTGATTTTGCCCGGATTTGGTGTTATTAGTCATATTTGTTTGA
GGATTAGTTCTAATTTGGATGTTTTTGGGTTTTATGGGTTGTTGTTTGCTATGTTTTCTATAGTGTGTTT
AGGTAGTAGTGTTTGGGGACATCATATGTTTACTGTTGGATTAGATGTGAAGACTGCTGTTTTTTTTAG
TTCTGTTACTATGATTATAGGTGTTCCTACTGGTATAAAGGTGTTTACTTGGTTGTATATGTTATTGAAT
TCTAATGTTAATGCTAGTGATCCTGTTTTGTGGTGGGTTATTTCTTTTATAGTTTTATTTACGTTTGGGG
GCGTCACTGGTATAGTTTTGTCTGCTTGTGTGTTGGATAATGTTTTACATGATACTTGGTTTGTAGTGG
CTCATTTTCATTATGTTCTTTCTTTA

Primer regions, were shaded in grey.

CO1 Forward primer:

5'-TTTTTTGGGCATCCTGAGGTTTAT-3'

CO1 Reverse primer:

5'-TAAAGAAAGAACATAATGAAAATG-3´
